# Does osteosarcopenia shorten life?

**DOI:** 10.1590/1806-9282.20250442

**Published:** 2025-10-27

**Authors:** José Eduardo Nogueira Forni

**Affiliations:** 1 Faculdade de Medicina de São José do Rio Preto, Department of Orthopedics and Traumatology – São José do Rio Preto (SP), Brazil.

Osteosarcopenia is defined as the simultaneous occurrence of osteoporosis and sarcopenia. The incidence is highest in older adults, and the condition is characterized by low bone mineral density with an increased risk of fractures, along with a reduction in muscle mass that leads to a decline in muscle strength and power, which, in turn, increases the risk of falls and disability. Osteosarcopenia leads to chronic pain, hospitalization, and a reduction in quality of life, as well as an increased risk of mortality^1^.

Binkley and Buehring first defined this condition in 2009 for older patients with both osteoporosis and sarcopenia and warned of the risks of falls, disability, and fractures^2^.

The prevalence of osteosarcopenia varies among countries and geographical regions and depends on factors such as age, sex, and diagnostic criteria. However, the global rate among older people ranges from 5 to 40%^3^.

The identification of risk factors is fundamental to the prevention and treatment of osteosarcopenia. The risk is fivefold higher in women compared to men due to the decline in estradiol that occurs with menopause. Moreover, fat infiltration in muscles and bones causes the secretion of a hormone associated with proinflammatory cytokines, such as interleukin (IL)-6, tumor necrosis factor (TNF)-α, and IL-1, leading to the apoptosis of myocytes and osteocytes^4^.

This clinical condition involves the loss of muscle strength and impaired balance, which can lead to frequent falls and fractures, and may also be associated with cognitive decline^5^. The diagnosis is based on a detailed medical history complemented with strength tests and the sit-to-stand test, as well as exams such as bioimpedance, ultrasound, quantitative computed tomography, magnetic resonance, and the determination of bone mineral density^6^.

Non-pharmacological treatment involves resistance exercises for 20 min two to three times a week and nutritional support. Pharmacological treatment involves medication for chronic pain and high doses of vitamin D, along with calcium, antiresorptive agents, and the replacement of all deficient vitamins.

It is extremely important for healthcare providers—especially clinicians who treat patients with chronic pain—to be attentive to this diagnosis and initiate early treatment to avoid the occurrence of serious complications, including death. The author recommends that this serious condition be included in the curricula of undergraduate courses in medicine as well as graduate programs in the health sciences.

## Data Availability

The datasets generated and/or analyzed during the current study are available from the corresponding author upon reasonable request.
